# Skeletal Muscle Homeostasis in Duchenne Muscular Dystrophy: Modulating Autophagy as a Promising Therapeutic Strategy

**DOI:** 10.3389/fnagi.2014.00188

**Published:** 2014-07-24

**Authors:** Clara De Palma, Cristiana Perrotta, Paolo Pellegrino, Emilio Clementi, Davide Cervia

**Affiliations:** ^1^Unit of Clinical Pharmacology, Department of Biomedical and Clinical Sciences “L. Sacco”, National Research Council-Institute of Neuroscience, University Hospital “L. Sacco”, University of Milan, Milan, Italy; ^2^Scientific Institute IRCCS Eugenio Medea, Bosisio Parini, Italy; ^3^Department for Innovation in Biological, Agro-Food and Forest Systems, University of Tuscia, Viterbo, Italy

**Keywords:** skeletal muscle, Duchenne muscular dystrophy, muscle wasting, autophagy, Akt–mTOR axis, *mdx* mice, therapeutic target

## Abstract

Muscular dystrophies are a group of genetic and heterogeneous neuromuscular disorders characterized by the primary wasting of skeletal muscle. In Duchenne muscular dystrophy (DMD), the most severe form of these diseases, the mutations in the dystrophin gene lead to muscle weakness and wasting, exhaustion of muscular regenerative capacity, and chronic local inflammation leading to substitution of myofibers by connective and adipose tissue. DMD patients suffer from continuous and progressive skeletal muscle damage followed by complete paralysis and death, usually by respiratory and/or cardiac failure. No cure is yet available, but several therapeutic approaches aiming at reversing the ongoing degeneration have been investigated in preclinical and clinical settings. Autophagy is an important proteolytic system of the cell and has a crucial role in the removal of proteins, aggregates, and organelles. Autophagy is constantly active in skeletal muscle and its role in tissue homeostasis is complex: at high levels, it can be detrimental and contribute to muscle wasting; at low levels, it can cause weakness and muscle degeneration, due to the unchecked accumulation of damaged proteins and organelles. The causal relationship between DMD pathogenesis and dysfunctional autophagy has been recently investigated. At molecular level, the Akt axis is one of the key dysregulated pathways, although the molecular events are not completely understood. The aim of this review is to describe and discuss the clinical relevance of the recent advances dissecting autophagy and its signaling pathway in DMD. The picture might pave the way for the development of interventions that are able to boost muscle growth and/or prevent muscle wasting.

## Pathological Features of Dystrophin Deficiency

Duchenne muscular dystrophy (DMD) is the most frequent and lethal form of muscular dystrophy, affecting 1 out of 3,500 newborns (Govoni et al., 2013). DMD is an X-linked disorder caused by mutations of the largest gene of the human genome, the dystrophin gene. So far, more than 4,700 mutations have been identified and classified as deletions (65.8%), duplications (13.6%), and point mutations (micro-insertions, microdeletions, and nonsense, missense, and splicing mutations; 20.6%) (Magri et al., 2011). Mutations that change the reading frame of the gene generally result in premature stop codons with complete loss of dystrophin in DMD muscles. Other mutations can result in the generation of a smaller size-dystrophin or in a lesser amount of the protein being produced leading to the allelic dystrophinopathy disorder known as Becker muscular dystrophy, a disease milder than DMD, affecting 1 of 18,518 male births (Emery, 1991).

The diagnosis of DMD and Becker muscular dystrophy is based on careful analysis of the clinical features and confirmed by additional investigations including muscle biopsy and/or genetic testing. In particular, DMD is characterized by an early onset before 3 years of age, then ambulation loss occurs between 10 and 14 years of age, and finally death takes place between 20 and 30 years of age (Davies et al., 1988).

The most impaired muscle in DMD patients is the diaphragm, and its wasting is responsible for the respiratory failure (reduced wall and lung compliance, hypoventilation, hypercapnia, and hypoxemia) and thus death (Fayssoil et al., 2010; Mosqueira et al., 2013). DMD also affects cardiac muscle, and to a lesser extent, smooth muscle (Fayssoil et al., 2010; Mosqueira et al., 2013). More than 90% of DMD patients are affected by some degree of cardiomyopathy (Fayssoil et al., 2010; Mosqueira et al., 2013), which causes a progressive reduction in the ejection fraction, which may hesitate in heart failure with or without concomitant arrhythmias (Jefferies et al., 2005). The onset of cardiomyopathy is variable, starting from 18 years of age in DMD patients (Spurney, 2011; Politano and Nigro, 2012). Cardiac decline is observed also in Becker muscular dystrophy patients, which progresses faster than skeletal muscle decline (Bushby et al., 2010).

Functionally, dystrophic skeletal muscles are more susceptible to eccentric contraction than the healthy ones (Lynch et al., 2000). In response to this type of injury, altered muscle fibers undergo repeated cycles of necrosis and regeneration, during which satellite cells, the primary myogenic precursor cells of the muscle, activate muscle regeneration process (Le Grand and Rudnicki, 2007; Biressi and Rando, 2010). However, the regenerative process is rather inefficient and the repeated cycles of necrosis and regeneration lead to satellite cell depletion and gradual replacement of muscle by fat and connective tissue; this process is responsible for progressive muscle wasting and weakness. This may be attributed to a great extent to the loss of dystrophin.

Dystrophin is essential for maintenance of muscle membrane integrity; it is a scaffolding protein that recruits other structural and signaling proteins to the sarcolemma, forming a well-organized multimeric dystrophin-associated glycoprotein complex. The dystrophin-associated glycoprotein complex is composed of several transmembrane and peripheral proteins, depending on the tissue, in particular it encloses different sarcoplasmic proteins [α- and β-dystrobrevins, α1-, β1-, β2-, γ1-, and γ2-syntrophins, and neuronal nitric oxide synthase (nNOS)], transmembrane proteins (β-dystroglycan, α-, β-, γ-, δ-, ε-, and ζ-sarcoglycans, sarcospan, and caveolin-3), and extracellular proteins (α-dystroglycan and laminin). Dystrophin-associated glycoprotein complex plays a key role in the mechanical stabilization of the sarcolemma during muscle contractions (Straub and Campbell, 1997), and acts as a scaffold for different proteins implicated in intracellular signaling. The proteins inside dystrophin-associated glycoprotein complex are tightly connected to each other and abnormalities affecting one of these proteins lead to changes in the others impairing muscle function. Of importance, the disruption of the dystrophin-associated glycoprotein complex in DMD also causes the delocalization of the nNOS from sarcolemma, leading to a reduced generation of NO. NO is important in skeletal muscle physiology as it controls excitation–contraction coupling and energy balance (De Palma and Clementi, 2012). The loss of NO-dependent signaling pathways contributes significantly to impair muscle bulk and force generation with increased fatigability (De Palma and Clementi, 2012). Moreover, the loss of sarcolemmal nNOS contributes to increase the muscle fibers susceptibility to ischemia during exercise, and to mechanical damage resulting in a focal muscle injury, dependent on muscle use (Thomas, 2013).

### Mouse models

In DMD, different mouse models with mutations in dystrophin gene have been generated. The *mdx* mouse on C57BL/10ScSn genetic background (C57BL/10ScSn-*Dmd^mdx^*, here referred as “*mdx* mouse”) (Coulton et al., 1988) shows a point mutation in exon 23, generating an early stop codon and leading to the absence of full-length dystrophin (Willmann et al., 2009). Other models bearing different mutations have been also generated, as for instance *mdx*2cv, *mdx*3cv, *mdx*4cv, or *mdx*5cv mouse (Im et al., 1996), *mdx*52 (with mutation on exon 52; Araki et al., 1997), and *mdx*/utr^−/−^ (*mdx* mouse with additional knockout of utrophin) (Deconinck et al., 1997).

Among these models, the *mdx* mouse is the most commonly used (Nakamura and Takeda, 2011). The mutation on exon 23 is wide spread and affects one-third of DMD patients (Willmann et al., 2009). *mdx* mice have a shorter life compared to controls and display a decreased relative muscle force, whereas absolute muscle force is unaffected. Isolated muscles show a reduced force contraction, even if lacking standard condition of analysis is difficult to compare results from different groups.

Degeneration, regeneration, and necrosis are processes observed in young *mdx* mice (2–4 weeks) that result in increased number of regenerating-centronucleated fibers and in the heterogeneity of myofibers area (McGeachie et al., 1993). Necrotic fibers can be found at any age with very high frequency after 18 months (Nakamura and Takeda, 2011). Muscles undergo frequent cycles of necrosis/regeneration, associated with weakness and muscle loss. These cycles of regeneration lead to a muscle phenotype milder than the one observed in human patients. Fibrosis appears to be much less than in DMD patients, except for diaphragm muscle (Nakamura and Takeda, 2011). Although the *mdx* mouse diaphragm reproduces the degenerative changes of DMD, respiratory complications are visible only in 16-month-old mice (Stedman et al., 1991; Nakamura and Takeda, 2011). Muscle pathology can be worsened by forced exercise; this is a viable strategy to worsen the disease phenotype and better reveal the efficacy of new therapies.

Of importance, *mdx* mouse also displays cardiomyopathy, characterized by fibrosis and presence of necrosis and inflammation, thus sharing some aspects of DMD-patients cardiomyopathy and constitute a good model to investigate cardio-protective candidate molecules (Quinlan et al., 2004).

## Autophagy and Its Role in Skeletal Muscle Homeostasis

Autophagy is a crucial mechanism involved in the turnover of cell components both in constitutive and catabolic (stress, nutrient deprivation, cytokines, amino acids deprivation) conditions. Autophagy classically functions as a physiological process to degrade cytoplasmic components, protein aggregates, and/or organelles, and as a regulator of cellular architecture. Autophagy in mammals generally protects the cells from death and defective autophagy can be associated with several diseases including cancer, neurodegenerative diseases, infectious diseases, and metabolic diseases (Cervia et al., 2013; Jiang and Mizushima, 2014; Schneider and Cuervo, 2014).

So far, three different mechanisms of autophagy have been described: macroautophagy, microautophagy, and chaperone-mediated autophagy. The most part of the information on autophagy in skeletal muscle is about macroautophagy, a process characterized by membranes that grow in size to generate double membrane-structures termed autophagosome, that enclose organelles, portion of cytoplasm, or protein aggregates. In this process, small ubiquitin-like proteins are required for the formation of autophagosomes and they are covalently bound to phosphatidylethanolamine.

Autophagy was considered initially as a non-specific degradation mechanism, but over the years selective forms of autophagy have been identified. For instance, selective removal of organelles, such as mitochondria or peroxisomes occurs *via* specific types of autophagy, termed mitophagy or peroxophagy, respectively.

### Autophagic signaling

The molecular signaling pathway leading to autophagy is very complex and regulated by autophagy-related genes (Atgs), which are connected with the formation of autophagosomes (Hurley and Schulman, 2014). The protein products of Atgs are organized in five functional groups, namely: (i) the Unc-51-like kinase (Ulk):Atg13:FIP200 initiation complex (Ganley et al., 2009; Hosokawa et al., 2009); (ii) the beclin1:hVps34[phosphatidylinositol 3 (PI3) kinase]:Atg14L nucleation complex (Itakura et al., 2008); (iii) the PI3–phosphate-binding WIPI-1/2 complex (Proikas-Cezanne et al., 2004; Vergne et al., 2009); (iv) the Atg5–Atg12 conjugation complex activated by Atg7 (Mizushima et al., 1998); and (v) the Atg8 (LC3) conjugation system (Kabeya et al., 2000). These protein complexes participate at specific stages in the autophagic process: initiation, formation, elongation, and fusion (Mehrpour et al., 2010; Awan and Deng, 2014); they are also controlled by several other signaling pathways that fine tune autophagy to regulate the pace of autophagosome formation.

A key player in the control of autophagy is the mammalian target of rapamycin (mTOR), which together with raptor (regulatory associated protein of mTOR), G protein β-subunit-like protein (GβL) and proline-rich Akt substrate of 40 kDa (PRAS40) forms the multiprotein complex, mTORC1. The activity of mTOR, a negative regulator of autophagy, depends on several positive signals including normoxia, amino acid supply, high energy levels, or growth factors. Upon stimulation by growth factors or nutrients (glucose, amino acids), mTORC1 negatively regulates the macromolecular initiation complex Ulk:Atg13:FIP200 leading to autophagy suppression (Ganley et al., 2009; Hosokawa et al., 2009; Wong et al., 2013). Conversely, starvation and energy depletion, which stimulate autophagy, inhibit mTORC1, leading to the activation of Ulk (Jung et al., 2010; Kim et al., 2011; Mihaylova and Shaw, 2011).

The main signaling pathway controlling mTORC1 is the PI3 kinase/Akt pathway activated by the binding of growth factors or insulin to their cell surface receptors. Activated Akt in turn phosphorylates and inhibits the tuberous sclerosis complex 2 (TSC2), thus preventing the formation of the inhibitory TSC1/TSC2 heterodimer. This inhibition allows the small GTPase Rheb to activate directly mTORC1 (Long et al., 2005a,b; Huang and Manning, 2009) and to inhibit autophagy. Another signaling pathway controlling mTOR is the adenosine monophosphate-activated protein kinase (AMPK) pathway activated under energy-low conditions (Alers et al., 2012). Activated AMPK is known to regulate mTORC1 activity mainly through the phosphorylation and consequent activation of the negative regulator TSC2 (Inoki et al., 2003). However, the discovery that TSC2-deficient cells can respond to a decrease in energy levels has led to the investigation of additional mechanisms. Recently, it has been demonstrated that both mTORC1 and AMPK can act on the same substrate, Ulk1, with opposite effects: mTORC1 inhibits Ulk1 activation by phosphorylating Ser757; conversely, AMPK activates Ulk1 through its phosphorylation on Ser317 and Ser777 (Kim et al., 2011).

Additional proteins have been found to be associated with mTOR, i.e., rictor (rapamycin-insensitive companion of mTOR), GβL, SAPK-interacting protein 1 (SIN1), and protein observed with rictor (PROTOR), to form the multiprotein complex mTORC2 (Sarbassov et al., 2006). mTORC2 is involved in the phosphorylation and activation of Akt, thereby promoting its prosurvival action. Through this activation, mTOR determines the downregulation of the transcription factor Forkhead Box O3 (FoxO3), particularly important in muscle where it stimulates autophagy by enhancing the expression of genes including Atg12, Ulk1, Atg4b, and Gabarapl1 (Mammucari et al., 2007).

### Autophagy levels and skeletal muscle impairment

The role of autophagy in skeletal muscle has been investigated extensively (Sandri, 2010; Neel et al., 2013). Muscle mass represents 40–50% of the human body and is one of the most important sites for the regulation of metabolism. Excessive protein degradation in the skeletal muscle is detrimental for the economy of the body and can lead to death. Studies in mice with muscle-specific inactivation of autophagic genes have been used to identify the role of autophagy in muscles (Sandri, 2010; Neel et al., 2013). Briefly, ablation of Atg7 gene led to an altered muscle structure, with sarcomere disorganization and myofibers degeneration due to activation of an unfolded protein response, accumulation of abnormal mitochondria, enhanced oxidative stress, and increased concentration of polyubiquitinated proteins (Masiero et al., 2009). Altogether, these effects accounted for muscle weakness, atrophy, and other signs of myopathy such as an irregular distribution of fibers’ shape as well as fibers with a vacuolated cytosol. In general, the blocking of basal autophagy in muscle enhanced the accumulation of damaged and dysfunctional mitochondria, suggesting that mitophagy was impaired and critical to maintain muscle homeostasis (Masiero et al., 2009). Further, muscle-specific Atg5^−/−^ mice displayed an atrophic phenotype in the fibers of the fast type, associated to accumulation of autophagic substrates, for instance, ubiquitinated proteins (Raben et al., 2008). In particular, in fast muscles of Atg5^−/−^ mice, the size and density of lysosomes were increased and their distribution on the microtubules altered. Microtubules displayed a more linear organization compared to that of wild-type mice, demonstrating that autophagy is crucial also for the correct arrangement of microtubules (Raben et al., 2008).

Finally, in studies in which the transcription of FoxO3 was upregulated an enhanced autophagy–lysosome system was observed, especially during muscle wasting and the factor itself was enough to enhance autophagy process and to trigger atrophy (Mammucari et al., 2007). Noteworthy, a recent study showed that mice lacking the nutrient-deprivation autophagy factor-1, displayed muscle weakness, associated with an increased autophagy, dysregulation of calcium and accumulation of enlarged mitochondria (Chang et al., 2012).

The lesson from these results is complex and indicates a dual role for autophagy in skeletal muscle homeostasis: at high levels, it can be detrimental and contribute to muscle atrophy; at low levels, it can cause weakness and muscle degeneration, due to the unchecked accumulation of damaged proteins and organelles (Sandri, 2010; Sandri et al., 2013). Thus, a proper autophagic process is vital for both functional skeletal muscle, which controls the support and movement of the skeleton, and muscle metabolism (Neel et al., 2013).

## Deregulation of Autophagy in Duchenne Muscular Dystrophy

The fact that manipulation of animal models to dysregulate autophagy also leads to muscle pathology has prompted studies to investigate whether this process is altered in muscle diseases (Neel et al., 2013; Sandri et al., 2013). Of interest, our understanding on the role of detective autophagy in different forms of inherited muscular dystrophies, including Bethlem myopathy, Ullrich congenital muscular dystrophy, merosin-deficient congenital muscular dystrophy, and Emery–Dreifuss muscular dystrophy has emerged in the past 5 years (Sandri et al., 2013).

As initially postulated from indirect evidence, deficient autophagy was suggested to contribute to DMD pathogenesis. The presence of swollen and damaged mitochondria, protein aggregation, and distension of sarcoplasmic reticulum, which are cyto-pathological hallmarks of DMD, are often observed when autophagy is impaired (Culligan et al., 2002; Zhao et al., 2007). In addition, activation of Akt was significantly higher in muscles from *mdx* mice and dystrophin-deficient primary myotubes, thereby suggesting a defective autophagic process (Dogra et al., 2006; Peter and Crosbie, 2006). In line with these observations, DMD patients were found to exhibit a similar pattern of Akt activation (Peter and Crosbie, 2006).

The causal relationship between DMD pathogenesis and dysfunctional autophagy has been investigated more recently in studies addressing this issue specifically. A severe impairment of autophagy was indeed demonstrated by biochemical and ultrastructural analyses in muscles from patients affected by DMD and *mdx* mice. In particular, these muscles display a significant reduction in the lipidated form of the protein LC3, which is a common marker of autophagy induction (De Palma et al., 2012; Bibee et al., 2014). The reduction in lipidated LC3 has been found to be accompanied by clear signs of impaired autophagy at the ultrastructural level, i.e., by the presence of damaged organelles, the increase in the signaling adaptor p62 protein (a marker inversely correlated with autophagic flux), and the decrease of Bnip3, a mitochondrial protein, which recruits LC3 to mitochondria (De Palma et al., 2012; Bibee et al., 2014). Recent studies have also shown a role for the TNF receptor-associated factor 6 (TRAF6) as an important regulator of autophagy. In skeletal muscle of *mdx* mice, the activity of TRAF6 is increased and its absence correlates with a reduced autophagy (Hindi et al., 2014). Strikingly, this study also reported that the inhibition of TRAF6 signaling deteriorates muscle pathology at later stages of disease progression. It has been thus hypothesized that the initial inhibition of autophagy in young *mdx* mice in the absence of TRAF6 gene may be a protective mechanism to preserve skeletal muscle mass. Autophagy emerges then as an essential process for the clearance of defunct cellular organelles and a continued inhibition of autophagy exaggerates dystrophic phenotype (Hindi et al., 2014). The beneficial effects of activating autophagy in *mdx* mice have been also confirmed by overexpressing peroxisome proliferator-activated receptor γ coactivator 1α (PGC-1a), a transcriptional coactivator, which is a powerful mediator of muscle plasticity (Hollinger et al., 2013) and by pharmacological treatment with an agonist drug against the energy sensor AMPK, i.e., 5-aminoimidazole-4-carboxamide-1-β-d-ribofuranoside (AICAR) (Pauly et al., 2012). In particular, AICAR treatment led to a significant activation of the autophagy, as indicated by the characteristic biochemical changes of increased lipidated LC3 content, an upregulation of other prototypical autophagy-associated proteins, and to significant improvements in both muscle structure and maximum force-generating capacity (Pauly et al., 2012).

From a functional point of view, it has been recently shown that voluntary exercise in *mdx* mice enhances markers of autophagy to or above the levels observed in healthy mice, thus suggesting that beneficial autophagy can be induced by exercise (Hulmi et al., 2013). In agreement with these data, fasting induced autophagy in wild-type and *mdx* mice diaphragm (Spitali et al., 2013). Interestingly in *mdx* mice, the ability of upregulating autophagy appears restricted to certain muscles: *tibialis anterior* muscle of *mdx* mice are unable to enhance autophagy in response to fasting, which induces autophagy in *tibialis anterior* muscle of wild-type mice.

### Molecular characteristics

**Figure 1:**
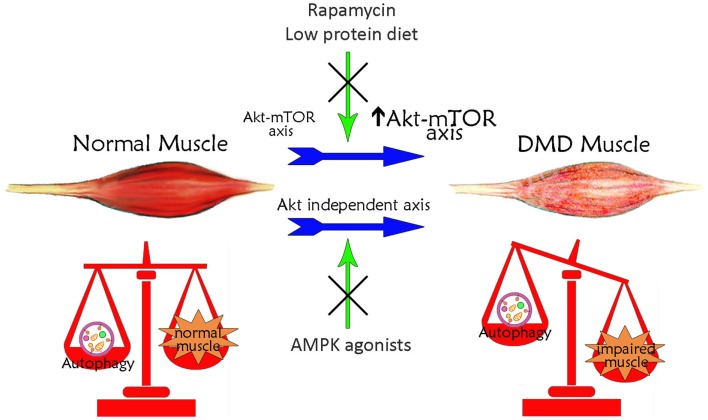
**Schematic illustration of the correlation between autophagy and Duchenne muscular dystrophy**. Basal autophagy levels are required for muscle homeostasis and for the maintenance of healthy myofibers. In DMD, muscle autophagy is impaired contributing to muscle degeneration. This autophagy inhibition is dependent on the iper-activation of Akt–mTOR axis. Treatments with rapamycin or low-protein diet, acting on Akt pathway, restore autophagy ameliorating DMD muscle phenotype and function. The impaired autophagy may also occur independently of Akt. Treatments with AMPK agonists, which increase AMPK activation, counteract the Akt-independent axes enhancing autophagy and inducing a positive effect in DMD muscle.

The defect in autophagy of *mdx* mouse muscles is accompanied by persistent activation of the Akt–mTOR axis (Figure 1) and its related autophagy-inhibiting pathways, and the concomitant downregulation of several autophagy-inducing genes (Dogra et al., 2006; Peter and Crosbie, 2006; De Palma et al., 2012). In particular, Akt activation has been established to occur at very early, pre-necrotic stages of disease pathogenesis, with a progressive increase with disease worsening (Peter and Crosbie, 2006). This high activation of Akt stimulates in turn the mTOR-dependent pathways whereas the mTOR-independent axis is not significantly altered (Dogra et al., 2006; Peter and Crosbie, 2006). The involvement of the mTOR axis in the pathogenesis of *mdx* mice is demonstrated also by the effects of the mTOR-targeting, immunosuppressant drug rapamycin. Oral or injected rapamycin treatment has been shown to improve histopathological features of dystrophy (Eghtesad et al., 2011) and the treatment with rapamycin-loaded nanoparticles (RNPs) has been shown to increase skeletal muscle strength in both young and adult mice concomitantly to an increased mTOR-dependent autophagy (Bibee et al., 2014). Consistently, *mdx* mice treatment with a long-term low-protein diet reactivates autophagy in muscle fibers, with increased lipidation of LC3, reduced levels of p62, normalization of Akt and mTOR signaling, a reduced accumulation of damaged organelles, and a significant recovery of muscle inflammation, fibrosis, myofiber damage and muscle function (De Palma et al., 2012). Since the long-term low-protein diet was shown to preserve the regenerating ability of *mdx* mouse muscle, it is tempting to speculate that physiological levels of autophagy maintain the number and function of myogenic precursor cells.

The picture is not completely clear and some controversies still exist and issues remain to be solved. Spitali et al. (2013) reported that autophagy levels and Akt axis activation in *mdx* mice is similar to wild-type control mice. Likewise, a study on Akt–mTOR axis activation in very old *mdx* mice correlated the advancing of age with a reduction in mTOR signaling in dystrophic muscles (Mouisel et al., 2010). In another study, however, it was also reported that mTOR activation is increased by age in diaphragm muscle of wild-type mice, but not in *mdx* mice, while similar levels of mTOR activation were found in *tibialis anterior* muscles (Eghtesad et al., 2011). Although these data are apparently difficult to reconcile, different confounding factors may explain them, as for instance the fact that *mdx* mice undergo extensive muscle regeneration between 6 and 12 weeks. Furthermore, the fact that the regulation of autophagy in *mdx* mice was indicated to differ depending on the muscle type (Spitali et al., 2013) should be taken into consideration, with the glycolytic muscles showing a process of vesicles formation significantly greater than oxidative muscles (Mizushima et al., 2004).

Autophagy can be modulated in *mdx* mice also through mTOR-independent mechanisms (Figure 1). Chronic AICAR administration induced autophagy in *mdx* muscles, with a significant upregulation of Ulk1 and an increased expression of Bnip3 in the absence of mTOR inhibition (Pauly et al., 2012). In addition, it has been suggested that TRAF6 regulation of Akt signaling is independent of TRAF6 regulation of autophagy (Hindi et al., 2014). The evidence for potentially augmented autophagic signaling may thus be suggestive of an adaptive response to attempt to rid dystrophic myofibers of defective and/or detrimental constituents, such as dysfunctional mitochondria (Ljubicic and Jasmin, 2013). The complex and apparently contrasting evidence on the role of authophagy in *mdx* mice suggests that the autophagic machinery in these mice is more complex than in wild-type mice and that a systematic analysis taking into account the variable of time has to be undertaken to clarify each single aspect in distinct muscles and during disease progression.

## Therapeutic Implications of Corrective Autophagy in Duchenne Muscular Dystrophy

At present, there is no satisfactory therapy for DMD. The treatment with corticosteroids is the actual gold standard. Treatment with glucocorticoids significantly delays the impairment of muscle force and function, extends ambulation period, and retards the onset of pulmonary failure, cardiomyopathy, and scoliosis (Bushby et al., 2010). These effects, however, are often only temporary and associated with severe side effects. More than 25% of patients are not treated with glucocorticoids due to adverse effects, such as obesity, immune suppression, bone demineralization, associated with negative behavioral changes, or lack of response (Bushby et al., 2010). This strongly indicates an urgent requirement of new clinical intervention for DMD patients.

The genetic approaches [exon skipping, viral vector-mediated gene delivery, and cell therapy (Pichavant et al., 2011; Mendell et al., 2012)], currently being investigated, show some degree of success and some of them have been recently granted orphan status by either the European Medicines Agency or the Food and Drug Administration (http://orphandruganaut.wordpress.com/2013/12/14/duchenne-muscular-dystrophy-2013-fda-orphan-drug-designations-2/). They, however, are directed to specific subsets of population and cannot restore fully the damage already caused by the disease to the muscle. Alternative strategies are therefore needed and to this end identification of suitable therapeutic targets is necessary.

In this context, pharmacological modulation of autophagy can be considered a possible strategy aimed at delaying muscle degeneration (Figure 1). As mentioned above, the AICAR activating AMPK, ameliorated phenotypic and functional features of dystrophic muscle (Pauly et al., 2012). AMPK pharmacological agonists exist; they are clinically approved and AICAR has been tested for ischemic damage in the heart and used in clinical trial for metabolic disorders (Ljubicic and Jasmin, 2013). The beneficial effects of AICAR in dystrophic muscle could be due to a well-known effect in stimulating the slow, oxidative phenotype (Ljubicic et al., 2011). It could also be due to induction of autophagic pathways, since AICAR stimulates the removal of damaged mitochondria via mitophagy (Pauly et al., 2012).

A second pharmacological approach that targets autophagy has been proposed by Bibee et al. (2014) in which RNPs were used to successfully enhance grip strength and the left-ventricular ejection fraction in *mdx* mice, ameliorating both physical and cardiac performances. These effects with RNPs have been achieved after administration of only eight doses of RNPs, with the final dose within the range of recommended oral doses for immune-suppressive therapy in patients. Interestingly, oral administration of rapamycin at pharmacological doses was of no efficacy on muscle strength. It appears that the way RNPs worked was via autophagy induction, dependent on nanoparticles delivery of the drug: rapamycin was locally delivered at high concentration thus becoming able to trigger autophagy (Bibee et al., 2014). Interestingly, at least in the *mdx* mice also corticosteroids induce autophagy and this may contribute to explain their beneficial effects.

Autophagy cannot certainly be envisaged as a stand-alone therapeutic option. Low-protein diet regimen has been recently hypothesized to be safely usable in the treatment of DMD patients in combination with pharmacological treatment and cell and gene therapies (De Palma et al., 2012). RPNs and low-protein diet might also be used in combination with corticosteroids to act as steroids sparing drugs, reducing their toxic effects. Low-protein diet has been demonstrated safe and useful also in Col6a1-deficient mouse, considered an animal model of Bethlem myopathy (Grumati et al., 2010). Of interest, a very recent clinical study showed a clear induction of autophagy with a low-protein diet regimen in muscles from Becker and Ulrich patients (Merlini and Nishino, 2014), further suggesting that also in humans a strategy of autophagy reactivation is applicable.

## Conclusion

Autophagy has emerged as a key process whose dysregulation contributes to the pathogenesis of several muscular dystrophies. The relevance of this process is that its normalization by pharmacological approaches leads to an amelioration of the dystrophic phenotype. While drugs targeting autophagy have good perspective in terms of therapy, we still need to refine them, by identifying appropriate targets in the autophagic pathway against which to design selected modulating drugs.

## Conflict of Interest Statement

The authors declare that the research was conducted in the absence of any commercial or financial relationships that could be construed as a potential conflict of interest.
